# DACH1 inhibits lung adenocarcinoma invasion and tumor growth by repressing CXCL5 signaling

**DOI:** 10.18632/oncotarget.3463

**Published:** 2015-02-28

**Authors:** Na Han, Xun Yuan, Hua Wu, Hanxiao Xu, Qian Chu, Mingzhou Guo, Shiying Yu, Yuan Chen, Kongming Wu

**Affiliations:** ^1^ Department of Oncology, Tongji Hospital of Tongji Medical College, Huazhong University of Science and Technology, Wuhan, P.R. China; ^2^ Department of Gastroenterology & Hepatology, Chinese PLA General Hospital, Beijing, P.R. China

**Keywords:** DACH1, Non-small cell lung cancer, invasion, tumor growth, CXCL5

## Abstract

Whole-genome and transcriptome sequencing of non-small cell lung cancer (NSCLC) identified that DACH1, is a human homolog of drosophila gene *dac*, is involved in NSCLC. Here we showed that expression of DACH1 was significantly decreased in human NSCLC tissues and DACH1 abundance was inversely correlated with tumor stages and grades. Restoration of DACH1 expression in NSCLC cells significantly reduced cellular proliferation, clone formation, migration and invasion *in vitro*, as well as tumor growth *in vivo*. Unbiased screen and functional study suggested that DACH1 mediated effects were dependent in part on suppression of CXCL5. There was an inverse correlation between DACH1 mRNA levels and CXCL5 in both lung cancer cell lines and human NSCLC tissues. Kaplan-Mier analysis of human NSCLC samples demonstrated that high DACH1 mRNA levels predicted favorable prognosis for relapse-free and overall survival. In agreement, high CXCL5 expression predicted a worse prognosis for survival.

## INTRODUCTION

DACH1 is a human homolog of drosophila gene *dac*, a key member of the Retinal Determination Gene Network (RDGN). RDGN is required for drosophila eye specification [1]. Through gene-specific recruitment of coactivator or corepressor that control precursor cell proliferation and survival, mamalian homologues, DACH1/SIX1/EYA network also determines the development of many organs, including lung [2, 3]. In this respect, transgenic model demonstrates that Six1 and Eya are required for maintaining epithelial progenitor cell, regulating epithelial branching, mesenchymal development as well as alveolarization during the saccular phase of lung morphogenesis [3,4]. Recently, more evidence indicates RDGN network is implicated in the development of human cancer, including breast and lung carcinoma [5-10]. Functional studies have identified DACH1 as a negative regulator of TGF-β and Wnt signaling to repress cancer cell migration and invasion [11,12]. DACH1 inhibits cell cycle progression and oncogenic transformation, as well as blocks paracrine signaling [13-15]. DACH1 attenuates the transcriptional activity of FOXM1b by a competitively binding to homologic DNA sequence [16]. In addition, DACH1 associates with the estrogen and androgen receptors (ER and AR) to regulate signal transduction and proliferation of breast and prostate cancer cells [17,18]. DACH1 inhibits EMT and tumor initiated cells in breast cancer and glioma [19-21]. DACH1 can directly associate with p53 and enhance its function in breast and lung cancer [10,22]. Inactivation of DACH1 in human cancer tissues was observed by mechanisms of gene deletion, mutation, or promoter hypermethylation [21,23]. Clinically, a decreased expression of DACH1 in breast and endometrial cancer correlates with tumor progression, poor differentiation [23] and predicts a short survival [13,24,25]. In human lung adenocarcinoma, the transcription factor SIX1 is upregulated during preinvasive to invasive transition and that results in EMT and conferring a more malignant phenotype [26]. In contrast, DACH1 expression was reduced in human NSCLC. Reexpression of DACH1 reduced colony formation and tumor growth in NSCLC cell lines via synergistic action with p53 [10]. However, the mechanism by which DACH1 regulates lung cancer growth is not fully understood. In this study, we analyzed the association between the DACH1 expression and clinic-pathological characteristics in NSCLC. Our result revealed low expressions of DACH1 predicted unfavorable prognosis for survival. CXCL5 was identified as a downstream target of DACH1-mediated repression of cell invasion and tumorigenesis. Conversely, high expressions of CXCL5 is associated with worse prognosis.

## RESULTS

### Decreased expression of DACH1 correlates with tumor progression and poor survival in NSCLC

**Figure 1:**
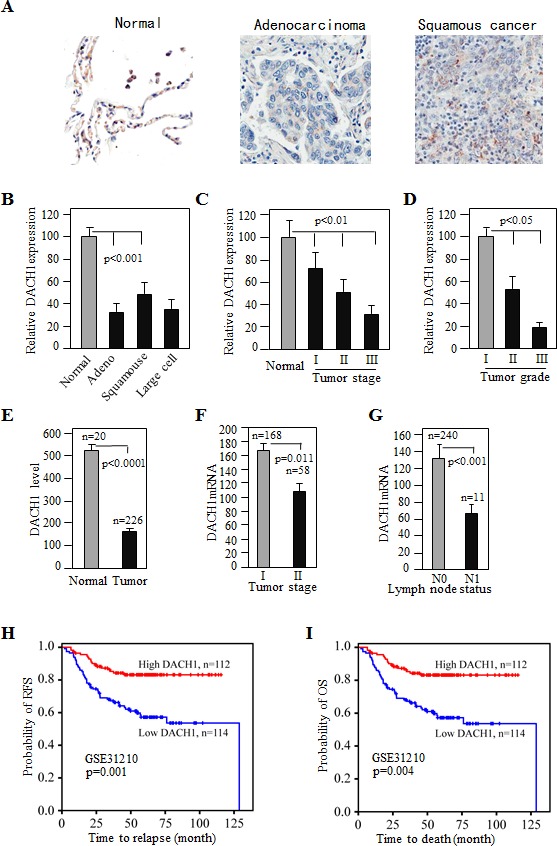
Decreased DACH1 expression in lung adenocarcinoma inversely correlated with tumor stage and related to survival (A) Representative images of DACH1 immunohistochemical stain of normal and lung cancer tissues. (B-D) Relative intensity of DACH1 protein in cancer tissues in relation to histological type, stage and grade. Quantitate mRNA expression of DACH1 in normal and cancer tissues (E), and DACH1 abundance in relation to tumor size (F) and lymph node status (G). Impact of DACH1 mRNA expression on relapse-free survival (H) and overall survival (I) revealed by Kaplan-Meier curves from dataset GSE31210.

To examine the expression of DACH1 in normal and cancer tissues of lung, immunohistochemical stain was performed on human lung cancer tissue arrays consisting of normal and different types of cancers. As previously reported, DACH1 was highly expressed in the nuclei of lung epithelial cells [10], but dramatically reduced in adenocarcinoma, squamous carcinoma, and large cell carcinoma (Fig. 1A, B). A semi-quantitive analysis revealed progressive loss of DACH1 expression in relation to tumor stage and histological grade (Fig. 1C, D). To determine whether loss of DACH1 occurred in the early stage, we analyzed a gene expression dataset of 226 primary lung adenocarcinoma with pathological stage I-II, the result showed that expression of DACH1 mRNA was reduced in tumor tissues (Fig. 1E) and inversely correlated with tumor stage (Fig. 1F). T2 tumor had much lower DACH1 expression than T1 tumor. In addition, tumor tissues with lymph node metastasis (N1) had much less DACH1 than those without metastasis (N0) (Fig. 1G). Furthermore survival analysis demonstrated that patients with high expression of DACH1 had 90% relapse-free survival (RFS) at 100 months follow-up, while RFS rate for patients in lower DACH1 expression group were only 60% (Fig. 1H). Similarly, patients with high expression of DACH1 were also associated with favorable overall survival (OS) than those with low expression, 95% vs 70% respectively (Fig. 1I). This results suggest that DACH1 expression was reduced in NSCLC tissues and inversely correlated with tumor progression. Low expression of DACH1 predicted worse prognosis.

### Restoration of DACH1 in NSCLC cells reduced cellular proliferation and tumor growth

**Figure 2:**
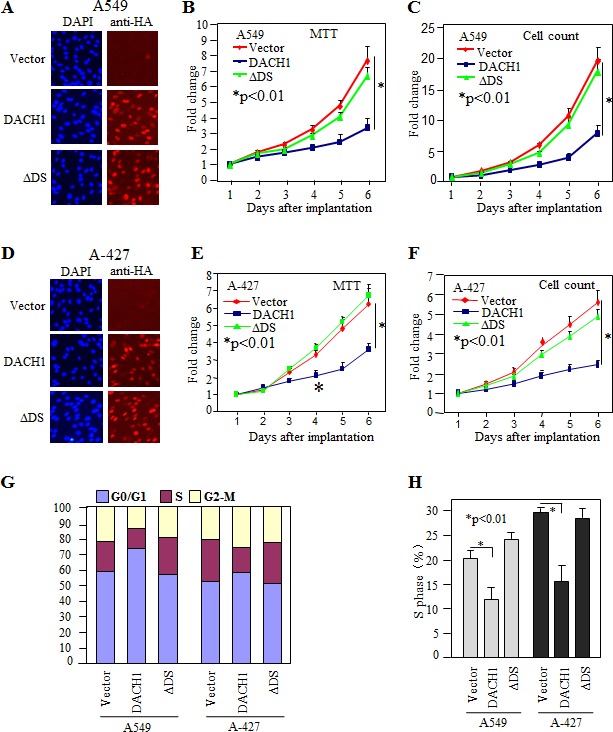
Ectopic expression of DACH1 inhibited cellular proliferation and cell cycle progression Stable expression of HA-tagged DACH1 in lung cancer cell lines A549 (A-C) and A-427 by fluororescent staining (D-F). Proliferation was evaluated by MTT assay (B, E) and cell counting (C, F). Cell cycle distribution evaluated by FACS (G, H).

**Figure 3:**
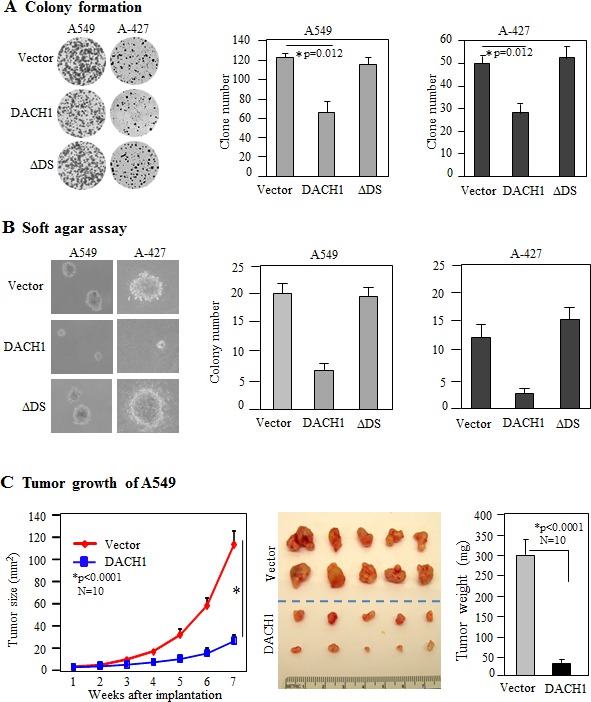
DACH1 inhibited clone formation and tumor growth *in vivo* (A) Representative images of contact-dependent clone formation and relative efficiency of 3 experiments for A549 and A-427. (B) Representative images of clone formation in soft agar and relative efficiency of 3 experiments for A549 and A-427. (C) Tumor growth curves of subcutaneously implanted A549 cells with or without DACH1 expression, representative images of dissected tumors and tumor weight.

In order to evaluate the role of DACH1 in NSCLC, we established DACH1 expressing lung adenocarcinoma cell line A-427 and A549 by lentivirus transduction. More than 95% of the cells were positive for ectopic expression of DACH1 as detected by fluorescent staining (Fig. 2A). Overexpression of DACH1 decreased A549 cellular proliferation assesssed by both MTT assay (Fig. 2B) and cell counting (Fig. 2C). The similar effects were observed in A-427 cell line (Fig. 2D-F). DS domain of DACH1 protein was required for inhibition of growth in breast and prostate cancer cells [20,24,26,27]. To examine whether this domain also plays an essential role in NSCLC, sublines expressing DACH1 without DS domian (ΔDS) were established. No significant effect on proliferation was observed by expression of ΔDS in both cell lines expressing. Cell cycle distribution analysis demonstrated that DACH1 decreased S phase ratio in comparision with vector control in both A-427 and A549 cells (Fig. 2G, H). Colony formation, especially contact-independent in soft agar, is a basic characteristic of transformed cells and partly represents the malignant potential and tumorigenicity. As we expected, ectopic expression of wild type DACH1 inhibited the clone number in both A-427 and A549 cells under contact-dependent growth. In contrast, equal expression of ΔDS had no repressive function (Fig. 3A). Colony formation in soft agar was also perfomed for both A-427 and A549 cells, the colony number was dramatically decreased in cells expressing wild type DACH1, whereas, there was no inhibitory effects by expressing ΔDS (Fig. 3B). To evaluate the role of DACH1 in tumorigenecity *in vivo*, A549 cells expressing either DACH1 or vector control were subcutaneously implanted into immunodeficient mice. As shown in (Fig. 3C), the dramatic decrease of tumor size was noticed in the animals inoculated with A549 cells expressing DACH1 compared with vector control. The average tumor weight decreased from 310mg in the control group to 40mg in the DACH1 group.

### DACH1 repressed NSCLC migration and invasion accompanied with decreased CXCL5 secretion

**Figure 4:**
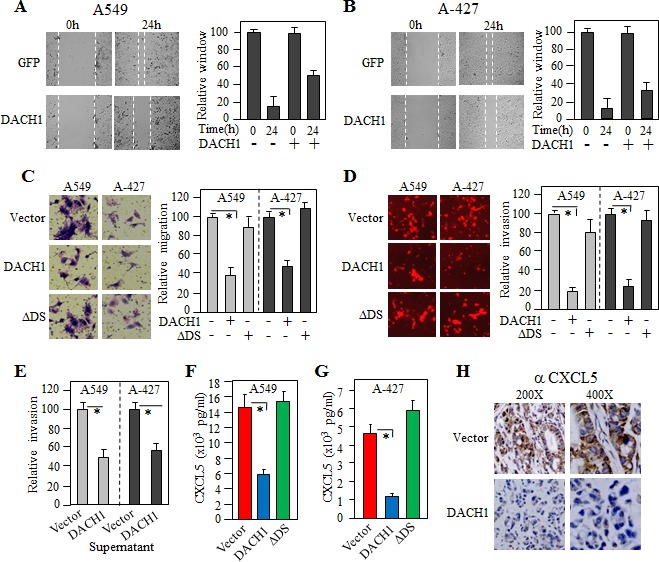
DACH1 inhibited lung cancer cell migration, invasion and CXCL5 secretion Representative images of wound-healing assay of A549 (A) and A-427 (B) cells, relative wound space was calculated on the right panel. (C) Representative images and quantitatively analysis of migrated A549 and A-427 cells expressing DACH1 or mutant ΔDS versus vector control. (D) Representative images and quantitatively analysis of invaded A549 and A-427 cells expressing DACH1, mutant ΔDS or vector control. (E) Migratory promoting effect of supernatant collected from A549 cells expressing DACH1 versus empty vector. Quantitative measurement of CXCL5 in the condition medium collected from A549 (F) or A-427 (G) by ELISA. (H) Representative immunohistochemical images of CXCL5 in implanted tumor from A549 expressing DACH1 versus control vector.

To investigate the function of DACH1 in cell movement, wound healing assay was performed. In both A549 and A-427 cells, expression of DACH1 significantly inhibited wound-healing process, suggeting a decreased cell mobility induced by DACH1 (Fig. 4A, B). To determine the migratory capacity of single cell, we performed transwell assay. In comparision to cells with vector, migrative cell number was reduced by more than 50% by expression of wild type DACH1, but not DS deleted mutant (ΔDS) (Fig. 4C). We further examined invasive capability using BD biocoated tumor invasion system. The inhibitory effect of DACH1 on invasion was even more dramatic compared to its effect on migration, decreased by more than 75% by DACH1 in comparision to vector. No significant effect was observed by ΔDS (Fig. 4D). We co-cultured A549 cells with conditional medium (CM) from either A549-vector or A549-DACH1, then observed migratory ability. The results indicated that incubation with supernatant from DACH1 expressing cells reduced cell motility, suggesting that CM in A549-DACH1 had less stimulative factors (Fig. 4E). Similar result was noticed in A-427 cells. To identify secreted factors, CM was employed to a cytokine antibody array spotted with 70 human chemokines/cytokines. Markedly reduced level of CXCL5 was noticed in CM from A549-DACH1 along with several other known cytokines, such as IL-8. To further confirm this results, we measured the CXCL5 levels by ELISA and found that there was more than 50% reduction of CXCL5 in both DACH1 expressing cell lines (Fig. 4F, G). To determine *in vivo* effect of DACH1 on CXCL5 expression, xenograft tumor tissues were immunohistochemically stained. The results showed that expression of CXCL5 was high in the cytoplasm of tumor cells expressing control vector, but very weak in tumor cells expressing DACH1 (Fig. 4H). Together, these results suggested that CXCL5 might be an important downstream target of DACH1 in repressing cell motility.

### CXCL5 rescued repression of cancer cell invasion and tumor formation by DACH1

**Figure 5:**
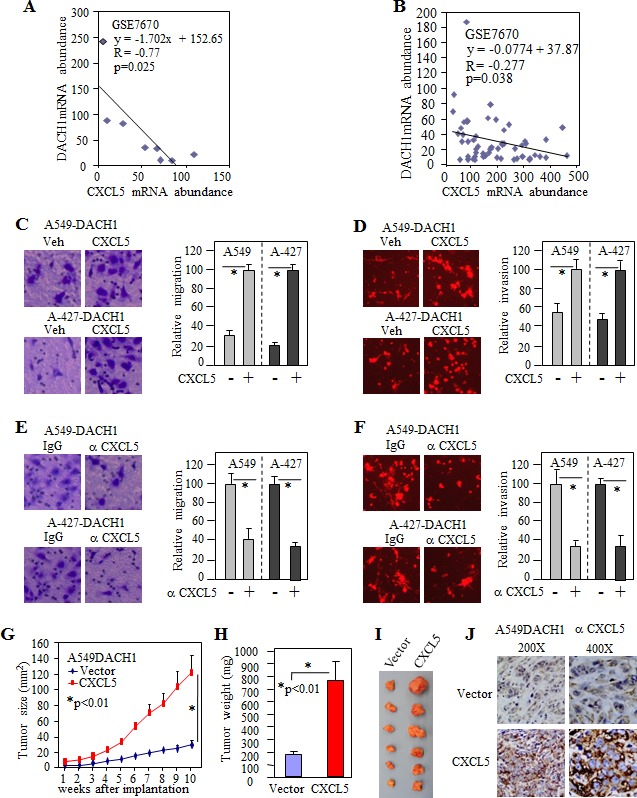
CXCL5 rescued invasion and antagonized tumor repression by DACH1 (A) The reciprocal mRNA expression between DACH1 and CXCL5 in normal lung epithelial cell line and 7 lung cancer cell lines from GSE7670. (B) The reciprocal mRNA expression between DACH1 and CXCL5 in NSCLC tissues from GSE7670. Addition of CXCL5 enhanced migration (C) and invasion (D) of A549 and A-427 cell expressing DACH1. Neutralized antibody to CXCL5 decreased migration (E) and invasion (F) of A549 and A-427 cells. Growth curve (G), weight (H) and representative images (I) of tumors from A549-DACH1 stable cells with ectopic CXCL5 expression versus vector control. (J) Representative immunohistochemical images of CXCL5 from A549-DACH1 cells with or without CXCL5 transduction.

To further explore the functional relationship between DACH1 and CXCL5, we searched lung cancer cell lines and tumor tissues database for mRNA expression. GEO dataset GSE7670 consisted of sixty-six affymetrix microarray information from 28 paired lung tumor and its adjacent normal tissues, 2 normal lung tissues and 8 cell lines, including immortalized human bronchial epithelial cell NL-20, adencarcinoma cell line A549, H1299, CL1-0 and large cell lung cancer cell line H661. The mRNA expression of DACH1 was inversely related to CXCL5 (R value = −0.77, p<0.025) (Fig. 5A) and such inverse relationship was also observed in normal and cancer tissues (Fig. 5B). To evaluate the role of CXCL5 in cell migration, purified CXCL5 was added into conditional medium, then migration and invasion was measured. Addition of CXCL5 to the medium enhanced cell migration and invasion in both A-427 and A549 cells (Fig. 5C, D). To further address the biological significance of CXCL5 in the CM, neutralized antibody to CXCL5 was added into the CM at final concentration of 1ng/ml before planting cells for motility assay. Antibody to CXCL5 significantly inhibited both migration and invasion in two cell lines at similar ratio (30%-40%) (Fig. 5E, F). To evaluate the role of CXCL5 in DACH1-mediated tumor growth, A549 cells stably expressing DACH1 (A549-DACH1) were co-transduced with lentivirus expressing CXCL5. In consist with (Fig. 3C), tumor derived from A549-DACH1 cells expressing vector control grown very slowly, but ectopic expresion of CXCL5 enhanced tumor growth to a rate similar to A549 parent cells (Fig. 5G). The average tumor weight were increased from 187mg to 735mg by CXCL5 (Fig. 5H, I). Immunohistochemical stain of xenograft tumor tissues confirmed strong ectopic expression of CXCL5 (Fig. 5J). Together, we provided an evidence that CXCL5 was a critical chemokine for DACH1-mediated repression of motility and tumor growth.

### CXCL5 expression correlated with tumor progression and might predict survival

**Figure 6:**
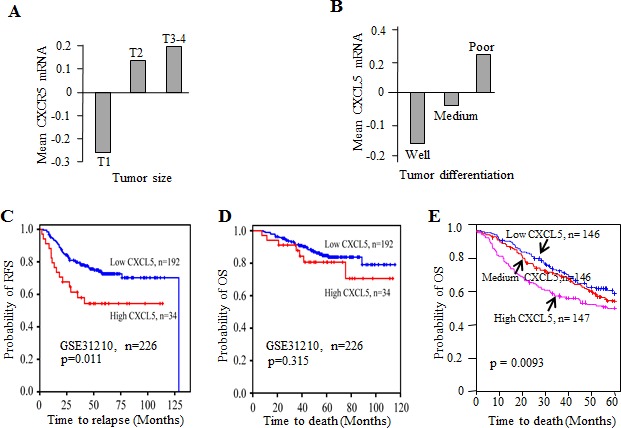
CXCL5 expression correlated with tumor stage and influenced survival in NSCLC Association of CXCL5 mRNA abundance with tumor size (A) and differentiation (B). Kaplan-Meier curves for relapse-free survival (C) and overall survival (D) from dataset GSE31210. (E) Kaplan-Meiercurves for OS for lung adenocarcinoma patients with low, medium or high expressions of CXCL5 mRNA.

To explore the potential value of CXCL5 expression as a biomarker in NSCLC, we analyzed GEO dataset GSE31210, which include 226 cases of pathological stage I-II lung adenocarcinomas with survival follow-up. CXCL5 mRNA abundance was positively related to tumor size with lowest expression in T1 and highest in T3-4 (Fig. 6A). Association of CXCL5 expression with histological differentiation status was also analyzed. Tumors with well differentiation expressed lowest level of CXCL5, whereas poorly differentiated tumors had highest expression of CXCL5 (Fig. 6B). In addition, we examined the prognostic value of CXCL5 mRNA for survival. 226 patients was divided as high versus low expression of CXCL5 based on mean value of CXCL5 mRNA. Kaplan-Meier survival curves showed that RFS rate at 100 months post operation was 70% versus 55% for patients with high expression of CXCL5 (Fig. 6C). The patients with low expression of CXCL5 had better OS, although not reaching statistical significance (Fig. 6D). We further analyzed mRNA profiles in another dataset with 439 lung adenocarcinomas, the patients were arbitrarily separated into three groups according to high, midium or low expression of CXCL5. The OS probabilities among the CXCL5 subgroups were statistically different (p=0.0093), indicating that CXCL5 expression was inversely correlated with overall survival (Fig. 6E).

## DISCUSSION

Recent findings supported the role of DACH1 as a novel tumor suppresor in several kinds of human cancers [5,12-25]. The link of DACH1 to lung cancer was initially uncovered by whole-genome and transcriptome sequencing of NSCLC samples. Genomic mutations and copy number loss of DACH1 gene were identified along with decreased DACH1 mRNA expression in NSCLC [27]. A subsequent study demonstrated the p53-dependent effect of DACH1 on proliferation and tumor gowth [10]. The current study further supported DACH1 as lung tumor suppressor. We found in NSCLC cell lines that the conserved DS domain was required for DACH-mediated repression, indicating a p53-independent alternative mechanism might be involved. By unbiased chemokine screen analysis, CXCL5 was identified as a novel target of DACH1. Functional analysis showed that CXCL5 played an essential role in DACH1-induced inhibition of cell migration *in vitro* and tumorigenesis *in vivo*. Importantly, there was an inverse relationship between the expression of DACH1 and CXCL5 in human lung cancer cell lines and NSCLC tissues.

CXCL5, also known as epithelial-derived neutrophil-activationg protein 78 (ENA78), is originally discovered as a potent chemoattractant and activator of neutrophil function. Via its receptor CXCR2, CXCL5 has been shown to regulate cell proliferation, cell migration and invasion [28]. Down-regulation of CXCL5 resulted in significantly decreased cell proliferation and migration. The tumorigenic potential was ablated by knock down CXCL5 [29]. In additin, CXCL5 promoted metastasis by regulating tumor-associated angiogenesis [30]. In deed, over-expression of CXCL5 increased tumor metastatic capacity.

CXCL5 expression could be regulated by several signaling pathways. DACH1 represses CXCL5 expression might be through crosstalk with those pathways. For instance, it has been reported that CXCL5 expression can be up-regulated by CREB and NF-kappaB in lung carcinogenesis [31]. In breast cancer, DACH1 has been shown to repress CXCL8 through blocking activation from AP1 and NF- kappaB binding sites [15] and inhibit cyclin D1 transcription though AP-1/CREB [13]. Therefore, we surmise that DACH1 associates with transcriptional factors to block CXCL5 activation. CXCL5 is well known to be activated by Ras signaling and the genetic ablation of its receptor, mCXCR2, reduced oncogenic Ras-driven tumorigenesis in mice [32]. In malignant peripheral nerve sheath tumors, activation of RAS repressed DACH1expression and inbibition of RAS signaling normalized the expresion of DACH1 [33]. Interestingly, we previously reported that DACH1 attenuated Ras-induced migration, invasion and CXCL8 secretion in breast cancer [15]. These studies suggest the existence of mutual regulation between Ras and DACH1. Thus, activated Ras in NSCLC could repress the expression of DACH1, in turn, reduced expression of DACH1 might enhance Ras activity, and that could lead to increased production of CXCL5. In addition, the FOXM1 is abundantly expressed in human NSCLCs and it transcriptionally induces the expression of genes essential for proliferation of tumor cells. In FOXM1 transgene model, elevated tumor formation was associated with persistent pulmonary inflammation, macrophage infiltration and increased expressions of CXCL5, CXCL1 and CCL3 [34]. It has been demonstrated that DACH1 antagonizes FOXM1 signaling through competitively binding to the conserved forkhead specific DNA sequence in breast cancer [16]. Therefore, we speculate that functional inactivation of DACH1 might disrupt the balance of DACH1-FOXM1, leading to the activation of CXCL5.

Besides activation of CXCL5 by oncogenes, CXCL5 has been shown to be repressed by tumor suppressor p53 [35]. We previously demonstrated that DACH1 associated with p53 protein to enhance p53 function in NSCLC [10]. In this regard, it is worthy to validate whether repression of CXCL5 by DACH1 depends on p53.

As to the clinical-pathological relevance of DACH1 in NSCLC, we observed that DACH1 abundance was inversely corelated with tumor stage, grade and metastasis, which is consistent with previous reports [10,12-14,23]. Thus far, only a handful of reports have explored the value of DACH1 as a molecular marker for cancer prognosis. We first reported that breast cancer patients with low DACH1 expression have a 40-month survival disadvantage [13]. Recently, Dr. Powe's study demonstrated that high expression of DACH1 predicted a better survival in luminal breast cancers subtype [24], further supporting the importance of DACH1 as a prognostic factor. Current analysis demonstrates that reduced expression of DACH1 is associated with unfavorable prognosis for RFS and OS. On the other hand, high expression of CXCL5 predicts worse prognosis for both RFS and OS. In deed, recent studies analyzed the effect of 18 out of the 23 differently expressed genes between the tumor and normal tissues on overall survival, only CXCL5 had significantly influence on overall and disease-free survival [36].

In summary, our study provides a functional link between DACH1 and CXCL5 in NSCLC. Considering the multiple functions of CXCL5 in terms of proliferation, invasion, inflamation and tumor-environment interaction, our results further enrich the understanding of the underlying mechanism by which DACH1 acts as a tumor suppressor.

## METHODS

### Patients and samples

The human lung cancer tissue arrays were purchased from Alenabio (Xi'an, China) and procedure was approved by the Ethical Committee of Tongji Hospital. There were 6 cases of normal lung tissues, 6 cases of cancer adjacent normal lung tissue, 57 cases of adenocarcinoma, 40 cases of squamous carcinoma, 31 cases of small cell carcinoma and 10 cases of large cell carcinoma. Tumor tissues were sub-grouped as grade I, well-differentiated; grade II, moderately differentiated; and grade III, poorly or undifferentiated. GSE31210 expression profiles consist of 226 cases with lung adenocarcinomas of pathological stage I-II [37].

### Immunohistochemistry and immunofluorescence stain

Tissue immunohistochemical stains were performed using a streptavidin-biotin technique and semi-quantified [14, 20]. The polyclonal antibody to DACH1(Proteintech, 10914-1) and to CXCL5 (Santa Cruz, sc-73930) were used by the published method [14]. The staining intensity was graded as follows: no staining, 0; weakly positive, 1; moderately positive, 2; and strongly positive, 3. Immunofluorescence staining for transduced DACH1 was detected by HA probe (Santa Crux, sc-7392).

### Cell culture and establishment of DACH1 stable cell lines

The human NSCLC cell lines A-427 and A549 were obtained from ATCC (Manassas, VA, USA) and were cultured in recommended medium supplemented with 10% fetal bovine serum. Human embryonic kidney 293T cells were maintained in DMEM containing 1% penicillin/streptomycin and supplemented with 10% FBS. Expressing vectors for wt DACH1 and ΔDS were described previously [13]. Lentivirus expression vector for human CXCL5 was purchased from DNASU plasmid repository. Lentivirus package and transduction were performed as manufacturer's recommended protocol. Stably expressions of DACH1 in transduced cells were confirmed by immunofluorescence stain. For selection of CXCL5 stable cells, transduced cells were treated with blasticidin at 5ug/ml for 2 weeks.

### Cell proliferation and colony formation assay

Cells expressing DACH1, ΔDS and vector control were seeded into 96-well plates in normal growth medium, and cell growth was measured by daily3-(4,5-dimethyl-thiazol-2-yl)-2,5-diphenyl-tetrazolium (MTT) assay as previously described [13,14]. For growth curve assay, cells were seeded into 12-well plates and serially counted for 6 to 7 days. For contact-dependent growth, 4 × 10^3^ cancer cells were plated in triplicate into 6cm dish and medium was changed every 3 days. The colonies were visualized by staining with 0.04% crystal violet in methanol for 1 hour [13]. For contact-independent growth, 4 × 10^3^cells suspended in 0.3% soft agar were plated on the surface of 0.5% soft agar in 6cm dish. After 2-weeks growth, colony with more than 30 cells was counted under 200X microscope [13, 14, 17].

### Cell cycle analysis

Cells were trypsinized and fixed in 70% ethanol, then processed by standard methods using propidium iodide staining of cellular DNA. Samples were analyzed on a FACScan flow cytometer (BD Biosciences). A minimum of 20,000 events for each sample were collected for statistical analysis of cell cycle distribution using ModFit version 2.0.

### Migration and invasion assay

For the wound healing assay, cells in 90% confluent were streaked with a 10 μl sterile pipette tip, washed with PBS, and replenished with serum-free DMEM. Images were captured using an inverted microscope at 12 and 24 hours after wounding. Migration assays were performed in transwell inserts with 8-μm pore (Corning Inc.; Corning, NY, USA). Serum-starved cells were trypsynized and counted. 2 × 10^5^ cells in 200 μl serum-free medium were placed into the upper chamber. The low chamber was filled with 500 μl of 10% FBS-DMEM. After 24 h, cells that migrated to the lower chamber were fixed and stained with hematoxylin. Invasion ability was quantitatively measured by BD coated invasion system.

### Tumor implantation study

A549 cells (2 × 10^5^) expressing either vector control or DACH1 were implanted subcutaneously to 6-week old ethymic male nude mice. The tumor growth was measured weekly for 4 to 5 weeks using a digital caliper. The isolated tumor mass was weighted after mice were sacrificed.

### Statistical analysis

All data were expressed as the mean±standard error. Statistical analyses between groups were calculated by student's t-test. P value <0.05 was considered stastically significant. Univariate cumulative survival analyses for relapse-free survival and overall survival were calculated by Kaplan-Meier curve using log rank tests.
